# Supplementary material for: Patterns of Intron Gain and Loss in Fungi
Source: PLoS Biol. 2004 Nov 30;2(12):e422. doi: 10.1371/journal.pbio.0020422 (PMC532390; doi:10.1371/journal.pbio.0020422)
Supplement: Table S1 — Also available at http://genes.mit.edu/NielsenEtAl/. (4.3 MB ZIP). [file pbio.0020422.st001.zip › NielsenEtAl/html/686.html]

AN1246.1.NCU07914.1.MG05063.1.FG03992.1


```
 CLUSTAL W (1.82) Multiple Sequence Alignments - Introns Inserted


Sequence 1: NCU07914.1	418 aa
Sequence 2: MG05063.1	418 aa
Sequence 3: FG03992.1	418 aa
Sequence 4: AN1246.1	421 aa
Alignment Length: 423 aa
Number Identitical Residues: 286 aa
Alignment Score (without introns) 12090


MG05063.1 	MSLSNKLSITDVDVKGKRVLIR0VDFNVPLD--DNKNITNNQRIAGAVPTIKHALDNGAK
NCU07914.1	MSLSNKLSIEDVDLKGKRVLIR0VDFNVPLD--AEKKVTNPQRIAGAIPTIKYALDHGAK
FG03992.1 	MSLSNKLSIADVDVKGKKVLIR0VDFNVPLD--ADKNITNNQRIVGALPTIKYALENGAK
AN1246.1  	MSLTSKLSITDVDLKDKRVLIR0VDFNVPLDKNDNTTITNPQRIVGALPTIKYAIDNGAK
          	***:.**** ***:*.*:**** ********.. :..:** ***.**:****:*:::***

MG05063.1 	TVILMSHLGRPNGSPNPKYSLKPVVSELEKLLGGKKVTFAPDCVGPEVEEIVNKAGEGDV
NCU07914.1	AVVLMSHLGRPDGKPNPKYSLKPVVPELEKLLG-KKVTFAPDCVGPEVEEIVNKADNGEV
FG03992.1 	SVILMSHLGRPNGSPNEKYSLKPVVAELEKLLS-KKVTFAPDCVGPEVEEIVNKAEDGSV
AN1246.1  	AVILMSHLGRPDGKKNPKYSLKPVVPKLKELLG-RDVIFTEDCVGPEVEETVNKASGGQV
          	:*:********:*. * ********.:*::**. :.* *: ********* ****  *.*

MG05063.1 	VLLENLRFHIEEEGSAKDKEGNKTKADKAKVEEFRKGLTKLGDIYIN1DAFGTAHRAHSS
NCU07914.1	ILLENLRFHIEEEGKGTDAEGNKVKADKAKVEEFRKNLTKLGDVYIN1DAFGTAHRAHSS
FG03992.1 	ILLENLRFHIEEEGSSKDKEGNKTKADKAQVEAFRKGLTALGDVYIN1DAFGTAHRAHSS
AN1246.1  	ILLENLRFHAEEEGSSKDADGNKVKADKDAVAQFRKGLTALGDIYIN1DAFGTAHRAHSS
          	:******** ****...* :***.****  *  ***.** ***:*** ************

MG05063.1 	MVGVDLPQKAAGFLMKKELDYFAQALESPKRPFLAILGGAKVSDKIQLIDNLLDKVNTLI
NCU07914.1	MVGIDLPVKAAGFLMKKELQYFAKVLESPQRPFLSILGGAKVSDKIQLIDNLLDKVNTLI
FG03992.1 	MVGVDLPQKASGFLVKKELEYFAKALENPQRPFLAILGGAKVSDKIQLIDNLLDKVNTLI
AN1246.1  	MVGVDLPQKASGFLVKKELEYFAKALEEPQRPFLAILGGSKVSDKIQLIDNLLPKVNSLI
          	***:*** **:***:****:***:.**.*:****:****:************* ***:**

MG05063.1 	VCGGMAFTFKKVLDNMAIGNSLFDEAGSKTVPELMKKAQEKGVKVVLPVDFITADKFAAD
NCU07914.1	VCGGMAFTFKKTLQNMPIGNSLFDEAGAKIVPDLVKKAEKNNVKLVLPVDFTIADKFDKD
FG03992.1 	ICGGMAFTFKKTLENVSIGNSLFDEAGSKTVGNLVEKAKQKGVKLVLPVDYITADKFDKD
AN1246.1  	ITGGMAFTFKKTLENVKIGSSLFDEAGSKIVGNIIEKAKKHNVKVVLPVDYVTADKFAAD
          	: *********.*:*: **.*******:* * ::::**:::.**:*****:  ****  *

MG05063.1 	AKTGSATDKEGIPEGWMGLDCGEKSVELFQSAIDESQTVLWNGPAGVFEFDAFAKGTKAT
NCU07914.1	ANTGYATDKDGIPDGWMGLDCGEESVKLFTQAINESQTILWNGPAGVFEFDKFAKGTKAT
FG03992.1 	ANTGYATDKDGIPDGWQGLDCGEESVKLYKEAIADAKTILWNGPAGVFEFEKFASGTKAT
AN1246.1  	AKTGYATDEQGIPDGYMGLDVGEKSVESYKQTIAESKTILWNGPPGVFEMEPFAKATKAT
          	*:** ***::***:*: *** **:**: : .:* :::*:*****.****:: **..****

MG05063.1 	LDAAVSGCGK-GKIVIIGGGDTATVAAKYGVEDKLSHVSTGGGASLELLEGKELPGVTAL
NCU07914.1	LDACVKAAEE-GRTVIIGGGDTATVAAKYGVEDKLSHVSTGGGASLELLEGKALPGVVAL
FG03992.1 	LDAVVDAVQKDGKIVIIGGGDTATVAKKYGVEDKISHVSTGGGASLELLEGKELPGVTAL
AN1246.1  	LDAAVAAVQN-GATVIIGGGDTATVAAKYGAEDKISHVSTGGGASLELLEGKELPGVAAL
          	*** * .  : *  ************ ***.***:***************** ****.**

MG05063.1 	SSK--
NCU07914.1	SERQ-
FG03992.1 	SSK--
AN1246.1  	SEKSK
          	*.:..
```
